# Performance of primary diagnostic monitors (PDMs) over time

**DOI:** 10.1002/acm2.12759

**Published:** 2019-12-13

**Authors:** Andres E. Ruuge, Yiming Gao, Yusuf E. Erdi

**Affiliations:** ^1^ Department of Medical Physics Memorial Sloan‐Kettering Cancer Center New York NY USA

**Keywords:** grayscale display function, primary diagnostic monitor, quality assurance

## Abstract

In this work, we evaluated the change of primary monitor characteristics in two consecutive years. Sixty‐six primary monitors were included in the analysis. The monitors were located at radiology physicians' offices and radiology reading rooms. All primary monitors were equipped with the manufacturer's built‐in photometers and connected to the BarcoMediCalQA web service for manual and automatic quality control measurements. External photometer/illuminance meter (RaySafe Solo Light) was used to measure the luminance values. Measured luminance values of the TG18LN1‐18 and TG18UNL80 test patterns were used to evaluate the primary monitors performance. In a comparison of the quality assurance (QA) measurement results for the same monitors that were performed within 2 years, the luminance of 25 displays remained statistically the same (*P* > 0.01). The luminance of 17 displays decreased (*P* < 0.01) in 2017 when compared with 2016, the luminance of 24 displays increased (*P* < 0.01) in 2017 when compared with 2016. For the annual measurements of the MLD in 2016 and 2017, 25 out of 66 displays showed a decrease of MLD values in 2017 compared with the same measurements in 2016 and 41 displays showed an increase of MLD in 2017. All tested primary displays had the MLD value less than 17.2%. The mean value of illuminance measured in 2016 was 5.8 lux ± 3.1 lux. In 2017, the mean value of illuminance measured was 8.7 lux ± 5.3 lux. Although it is expected that monitors luminance values will decrease over time, we found displays with increased luminance. This is possibly due to the multiple monitor calibrations that were performed between two annual monitor QA tests. Based on the findings of this work, more efficient display QA programs with a shorter time interval than 1 year are needed.

## 1 INTRODUCTION

Medical imaging plays an important role in a patient’s treatment. The ability of radiologists to recognize abnormal features on radiographic images depends on the display used to read the image. It is important that the image acquired using any modality is interpreted on a medical display that meets the set of requirements for image quality, medical regulations, and quality assurance.^1^, ^2^

The American Association of Physicists in Medicine (AAPM) TG‐18 report defines two classes of displays: primary displays are those used for interpretation of medical images and secondary displays include all other displays used in medicine.^2^ The ACR–AAPM–SIIM Technical Standard for Electronic Practice of Medical Imaging has provided basic recommendations regarding display performance characteristics for different display classifications.^3^ The Digital Imaging and Communication in Medicine (DICOM) Part 14: Grayscale Standard Display Function defines the calibration of primary displays.^4^

Quality assurance programs for displays used for the primary interpretation of medical images are required by some state and city regulators.^5^ Quality control (QC) measurements typically include an evaluation of display luminance characteristics such as the Grayscale Standard Display Function (GSDF), the luminance uniformity, and ambient light conditions of the display environment.

The GSDF curve is a luminance response curve of the display, which indicates that the display provides perceptual linearization for the human eyes.^4^ The characterization of the GSDF curve was performed by luminance measurements in steps of 18 gray levels both at acceptance and at annual testing of the display.^5^, ^6^ The luminance uniformity is assessed to ensure that the luminance in the corners and at the center of the display agree within a designated percentage. The luminance measurement is usually performed with an external photometer, which measures the intensity of visible light emitted from a unit area of the display’s surface. The results of the luminance measurements are part of the QC of the medical display. The illuminance on a display is the total luminous flux from all other light sources in the room and is evident on the viewing surface of the display. Illuminance is measured by an external photometer that that has been converted to an illuminance meter.

When assessing a display’s performance parameters, it is important to note that its luminance performance can change over time. This change may decrease a display’s overall performance, depending on its age and hours of usage.^6^, ^7^, ^8^, ^9^ The primary interest for this work is to determine how much the luminance level changes after 1 year of display usage.

## 2 METHODS

Measurements were performed on 66 primary displays of various models: MDCC‐6130 (35), MDCC‐6230 (5), MDCG‐10130 (2), MFGD 3420 (6), and MFGD 5421 (18) (Barco; Duluth, Georgia) as part of annual QA testing following New York City regulations.^5^ All measured displays were liquid crystal displays equipped with the manufacturer’s built‐in photometer (Barco, I‐Guard), which was connected to Barco MediCal QAWeb software for automatic built‐in QC service. A calibrated RaySafe Solo Light photometer (RaySafe; Billdal, Sweden) was used to perform manual luminance measurements using the TG‐18 test patterns. All displays were warmed up for at least 30 min before the luminance measurements were taken. The display output was left to stabilize after each switch of patterns, and then a stable reading on the luminance meter was obtained. To verify stability, several repeated recordings were made for each display to ensure that there was no drift in the photometer readings. The display performance data of the 66 primary displays was collected and analyzed for 2 years (2016 and 2017). The effect of changes in temperature and ambient light conditions on display luminance was assumed to be negligible because the locations of the primary displays in the diagnostic reading rooms remained the same over the year and ambient light conditions were controlled in those rooms.

### 2.1 Luminance measurements

Characterization of the GSDF curve was performed in steps of 18 gray levels, resulting in a total of 18 luminance measurements per year for each display. TG18LN1 through TG18LN18 test patterns were used to perform luminance measurements of the display.^2^, ^3^, ^5^ Each test pattern image in the series displayed a uniform background at a pixel value of 20% of the display’s maximum luminance, with a central square test region of specific luminance level occupying 10% of the image, allowing the repeatable positioning of the contact photometer during each of the 18 measurements. For data analysis, all displays were sorted by the year of initial use. For each display, the 18 luminance measurements in 2016 were compared with the 18 luminance measurements in 2017 using a paired t‐test. In addition, linear regression analysis was performed on all 1188 pairs of measurements to check whether human error or another unexpected error deviated any measurement from normal performance over the 2 years.

### 2.2 Display Uniformity

The Maximum Luminance Deviation (MLD) is described as the quantitative measure of display uniformity in the TG‐18 report.^2^ Luminance values were measured at five locations on the diagnostic display: the center and at each of the four corners using the TG18UNL80 test pattern. The MLD of a display was calculated using the following equation:

(1) $$\text{MLD}\left(\%\right)=200\;\times \;\left(L_{2}-L_{1}\right)/\left(L_{2}+L_{1}\right)$$

where *L*_2_ is the highest recorded luminance, and *L*_1_ is the lowest recorded luminance, regardless of the location on the display.^2^ MLD is a characteristic of a display that cannot be directly adjusted using the display calibration. The data of annual MLD changes for each studied display has been presented in this work. The difference between 2016 and 2017 MLD values was estimated for statistical significance by a Wilcoxon signed‐rank test, which is a suitable statistical test for dependent samples and is as efficient as a student t‐test for normal distributions.^12^

### 2.3 Illuminance measurements

In addition to diagnostic display luminance measurements, ambient illuminance was measured on each workstation display to confirm that the display’s ambient illuminance did not exceed 50 Lux, as is recommended in the ACR‐AAPM‐SIIM standard.^3^

The illuminance was measured using the same RaySafe Solo Light photometer configured for both luminance and illuminance measurements. The illuminance sensor was positioned in the center of the display facing outward while the primary display was turned off. The results of illuminance measurements for 2016 and 2017 were compared to evaluate the possible change in reading conditions. The difference between 2016 and 2017 was estimated for statistical significance by a Wilcoxon signed‐rank test.

## 3 RESULTS

### 3.1 Luminance measurements

All diagnostic displays analyzed in this study were sorted by the in‐service date and are presented in Table 1. All the displays were less than 8 years old at the time of the study.

**Table 1:** List of all measured primary display models (BARCO; Duluth, Georgia) included in the study and sorted by year of first use.

#	Model	In use since	#	Model	In use since	#	Model	In use since
1	MFGD 5421	09/2009	23	MFGD 5421	03/2012	45	**MDCC‐6130**	01/2014
2	MFGD 5421	09/2009	24	**MDCC‐6130**	03/2012	46	**MDCC‐6130**	01/2014
3	MFGD 5421	10/2009	25	**MDCC‐6130**	05/2012	47	**MDCC‐6130**	01/2014
4	MFGD 5421	10/2009	26	**MDCC‐6130**	05/2012	48	**MDCC‐6130**	01/2014
5	MFGD 5421	10/2009	27	**MDCC‐6130**	05/2012	49	**MDCC‐6130**	01/2014
6	MFGD 5421	10/2009	28	**MDCC‐6130**	05/2012	50	**MDCC‐6130**	01/2014
7	MFGD 3420	11/2009	29	MFGD 3420	06/2012	51	**MDCC‐6130**	01/2014
8	MFGD 3420	11/2009	30	MFGD 3420	06/2012	52	**MDCC‐6130**	02/2014
9	MFGD 5421	11/2009	31	**MDCC‐6130**	09/2012	53	**MDCC‐6130**	03/2014
10	MFGD 5421	11/2009	32	**MDCC‐6130**	11/2012	54	**MDCC‐6130**	03/2014
11	MFGD 5421	11/2009	33	**MDCC‐6130**	11/2012	55	**MDCC‐6130**	03/2014
12	MFGD 5421	11/2009	34	**MDCC‐6130**	08/2013	56	**MDCC‐6130**	03/2014
13	MFGD 5421	11/2009	35	**MDCC‐6130**	08/2013	57	**MDCC‐6130**	03/2014
14	MFGD 5421	11/2009	36	**MDCC‐6130**	08/2013	58	**MDCC‐6130**	03/2014
15	MFGD 5421	11/2009	37	**MDCC‐6130**	08/2013	59	**MDCC‐6130**	03/2014
16	MFGD 5421	11/2009	38	**MDCC‐6130**	08/2013	60	**MDCG‐10130**	05/2014
17	MFGD 3420	06/2011	39	**MDCC‐6130**	08/2013	61	**MDCG‐10130**	05/2014
18	MFGD 3420	06/2011	40	**MDCC‐6130**	08/2013	62	**MDCC‐6230**	06/2014
19	**MDCC‐6130**	03/2012	41	**MDCC‐6130**	08/2013	63	**MDCC‐6230**	11/2014
20	MFGD 5421	03/2012	42	**MDCC‐6130**	12/2013	64	**MDCC‐6230**	06/2015
21	MFGD 5421	03/2012	43	**MDCC‐6130**	12/2013	65	**MDCC‐6230**	06/2015
22	MFGD 5421	03/2012	44	**MDCC‐6130**	12/2013	66	**MDCC‐6230**	06/2015

Twenty‐four displays were calibrated for L_max_ = 500 cd/m^2^, and 42 displays (bold) were calibrated for L_max_ = 400 cd/m^2^.

Luminance measurements for each of the display paired t‐tests of 2016 and 2017 are presented in Table 2. Of the 66 displays, the luminance of 25 displays remained statistically the same (*P* > 0.01). The luminance of 17 displays decreased (*P* < 0.01) in 2017 when compared with 2016. Finally, the luminance of 24 displays increased (*P* < 0.01) in 2017 when compared with 2016.

**Table 2:** Paired t‐test (2016 luminance measurements vs 2017 measurements) results for all 66 monitors.

No change in Luminance *P* > 0.01		Luminance decreased in 2017 than in 2016, *P* < 0.01		Luminance increased in 2017 than in 2016, *P* < 0.01	
Monitor #	*P* Value	Monitor #	*P* Value	Monitor #	*P* Value
2	0.017	6	0.0003	1	0.0020
7	0.049	14	0.0095	3	0.0003
8	0.015	24	0.0005	4	0.0079
9	0.492	26	0.0023	5	0.0044
10	0.077	31	0.0001	15	0.0003
11	0.295	33	0.0002	17	0.0022
12	0.019	35	0.0032	20	0.0016
13	0.160	36	0.0023	21	0.0004
16	0.012	37	0.0090	22	0.0010
18	0.021	42	0.0027	23	0.0042
19	0.045	43	0.0066	27	0.0005
25	0.038	46	0.0074	28	0.0011
29	0.015	47	0.0028	34	0.0061
30	0.351	49	0.0002	41	0.0002
32	0.016	50	0.0006	44	0.0004
38	0.142	58	0.0011	45	0.0002
39	0.038	59	0.0002	51	0.0003
40	0.014	Count:	17	52	0.0013
48	0.316			53	0.0007
54	0.808			60	0.0007
55	0.096			61	0.0003
56	0.049			64	0.0038
57	0.029			65	0.0011
62	0.026			66	0.0003
63	0.117			Count:	24
Count:	25				

**Figure 1:**
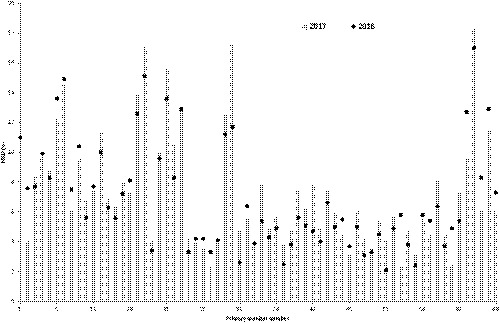
The linear regression with Pearson correlation coefficient = 0.999 (*P*‐value < 0.0001), where 1188 pairs of luminance observations (circles) are observed, shows measurements from 2016 and 2017 which are highly correlated and fall almost perfectly on the 45° line.

The 2016 and 2017 luminance measurement performance of the physicists was evaluated using simple linear regression. The scatterplot is presented in Fig. 1 where 1188 pairs of luminance observations are drawn as circles, showing that the measurements from the 2 years are highly correlated and almost fall perfectly on the 45‐degree line, with the Pearson correlation coefficient = 0.999 (*P*‐value < 0.0001).

### 3.2 Evaluation of Display Uniformity

**Figure 2:**
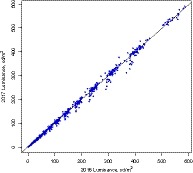
The results of annual measurements of the maximum luminance deviation (MLD) performed in 2016 (circles) and 2017 (columns). Twenty‐five out of 66 displays showed a decrease in MLD values in 2017 when compared with 2016, and 41 displays showed an increase in MLD in 2017. The maximum change in MLD was 11.7% in 2016 and 17.2% in 2017.

Figure 2 represents the results of annual measurements of the MLD in 2016 and 2017. Twenty‐five out of 66 displays showed a decrease of MLD values in 2017 compared with the same measurements in 2016, and 41 displays showed an increase of MLD in 2017. The maximum change in uniformity was 0.5% between 2016 and 2017. All tested primary displays had a MLD value less than 17.2%, well below the primary display QA standard of 30% or less.^5^

The Wilcoxon signed‐rank test with a significance level of *P*‐value = 0.01 showed the statistically insignificant differences in MLD measurements performed in 2016 and 2017 (*P*‐value = 0.56).

### 3.3 Illuminance measurements

The mean value of illuminance measured in 2016 was 5.8 lux ± 3.1 lux. In 2017, the mean value of illuminance measured was 8.7 lux ± 5.3 lux. The levels for both years met the American College of Radiology recommended less than 50 lux for a nonmammography diagnostic display radiology reading room.^3^

## 4 DISCUSSION

In recent years, there has been an increase in the number of primary displays deployed in our radiology clinics. The quality and accuracy of a radiologist’s reading depends on the performance of the display that he/she uses to review images and to perform diagnosis. Problems in display luminance, display uniformity, or ambient light could negatively affect image interpretation. To ensure that primary displays are performing according to their specifications, display performance parameters must be checked regularly as has been described in various publications.^7^, ^8^, ^9^, ^10^, ^11^

One of the objectives of a well‐established QC program for the primary displays is to follow and quantify any change of performance parameters over time, thereby detecting the problems that arise with the displays before they lead to clinical issues.

In this study, the changes in primary display performance parameters, such as luminance, were inconsistent across displays: they either stayed the same, decreased, or increased after 1 year of usage. Measurements of illuminance or luminance and their corresponding accuracies, are influenced by various parameters such as operational conditions and properties of light sources as well as characteristics of the applied photometers. However, the systemic error of the photometer (0.3%) could not fully account for all the different changes in the display performance.

The high correlation between 2016 and 2017 luminance measurements precluded human error or any random error from affecting the measurements. This finding suggests the performance of the physicists in the primary display tests was consistent through the 2 years.

The light sources around the primary displays were reviewed and modified to comply with the ambient light requirements of the standard at acceptance testing and were maintained over the 2 years.^3^ In addition, all measurements were performed after at least a 30‐min warm‐up of the displays based on the results of our previous work, which was performed to estimate the stability of luminance measurements for all tested primary display models.^9^ The results from that study confirmed that it is important to perform any luminance measurements after the display has been turned on for at least 30 min.^9^

Another source of inaccuracy in QC measurements of primary displays is that all displays in the program undergo automated calibration biannually. Using an internal photometer reading, display software calibrates the luminance level according to company specifications — in this case, 400 cd/m^2^ or 500 cd/m^2^, depending on the display model. During this process, display luminance levels are adjusted to meet the specifications. We found if annual QA was performed after this calibration, the external photometer would measure the adjusted level, which in turn would show changed luminance values and adjusted uniformity of the primary display in our preliminary investigations. However, the measurements included in this study did not control for the effect of the internal calibration of the displays. We suspect the automatic calibration of the displays is one of the sources of changes in the luminance measurements over the 2 years. Thus, future work will be to perform measurements before and after the automatic calibration of the displays to determine the effect of such calibration on luminance measurements.

The Wilcoxon signed‐rank test results of a display’s MLD (*P*‐value = 0.56) showed that the one‐year change in uniformity performance was not statistically significant. This finding suggests that the uniformity of the displays was not as sensitive as luminance to the automatic calibration of the displays.

We propose shorter time intervals of QC measurements based on the preliminary findings of the potential effect of automatic display calibration on the measurements for QA program improvement and research proposes if the resources allow such actions. Ideally, primary display QC programs can be performed at the time of the internal display calibration. As a result, fluctuation between the display calibration date and the display QC date would not affect the measured primary display parameters.

## 5 CONCLUSION

From this 2‐year investigation, it has been shown that the performance of several primary displays changes over time. Although it is unclear how much these performance changes impact the accuracy of clinical diagnosis, it is essential for radiology departments to develop QA programs and follow‐up with primary display performance at least annually and ideally even in shorter time intervals.

## CONFLICT OF INTEREST

The authors have no relevant conflicts of interest to disclose.
